# Super Divya to the rescue! Exploring Nurse Mentor Supervisor perceptions on a digital tool to support learning and engagement for simulation educators in Bihar, India

**DOI:** 10.1186/s12909-022-03270-5

**Published:** 2022-03-26

**Authors:** Anika Kalra, Manju Siju, Alisa Jenny, Hilary Spindler, Solange Madriz, Jami Baayd, Seema Handu, Rakesh Ghosh, Susanna Cohen, Dilys Walker

**Affiliations:** 1 Institute for Global Health Sciences, University of California San Francisco, 550 16th St, San Francisco, CA 94158 USA; 2 PRONTO India Foundation, State RMNCH+A Unit, C-16 Krishi Nagar, A.G. Colony, Patna, Bihar, 800013 India; 3 Department of Obstetrics and Gynecology, University of Utah, 30 North 1900 East, Salt Lake City, UT 84132 USA; 4 Department of Obstetrics, Gynecology and Reproductive Services, University of California San Francisco, 1001 Potrero Ave, San Francisco, CA 94110 USA

**Keywords:** Simulation training, Digital innovation, Virtual education, Obstetric and neonatal care, Nurse mentoring model, Qualitative study, Train-the-trainer

## Abstract

**Background:**

Since 2014, the Government of Bihar and CARE India have implemented a nurse mentoring program that utilizes PRONTO International’s simulation and team trainings to improve obstetric and neonatal care. Together they trained simulation educators known as Nurse Mentor Supervisors to conduct simulation trainings in rural health facilities across the state. Sustaining the knowledge and engagement of these simulation educators at a large-scale has proven difficult and resource intensive. To address this, the University of Utah with PRONTO International and with input from the University of California San Francisco, created an interactive, virtual education module based on a comic superhero named Super Divya to reinforce simulation educator concepts. This study examined the perceptions of Nurse Mentor Supervisors on Super Divya’s accessibility, usefulness, and potential after implementation of Super Divya: Origin Story.

**Methods:**

We conducted qualitative interviews with 17 Nurse Mentor Supervisors in Bihar, India. In light of the COVID-19 pandemic, interviews were conducted virtually via Zoom™ using a semi-structured interview guide in Hindi and English. Participants were identified with strict inclusion criteria and convenience sampling methods. Interviews were analyzed using a framework analysis.

**Results:**

Nurse Mentor Supervisors found Super Divya to be engaging, innovative, relatable, and useful in teaching tips and tricks for simulation training. Supervisors thought the platform was largely accessible with some concerns around internet connectivity and devices. The majority reacted positively to the idea of distributing Super Divya to other simulation educators in the nurse mentoring program and had suggestions for additional clinical and simulation educator training topics.

**Conclusions:**

This study demonstrates the potential of Super Divya to engage simulation educators in continuous education. At a time when virtual education is increasingly important and in-person training was halted by the COVID-19 pandemic, Super Divya engaged Supervisors in the nurse mentoring program. We have incorporated suggestions for improvement of Super Divya into future modules. Further research can help understand how knowledge from Super Divya can improve simulation facilitation skills and behaviors, and explore potential for reinforcing clinical skills via this platform.

**Ethical approval:**

This study was approved by the institutional review board at the University of California San Francisco (IRB # 20–29902).

**Supplementary Information:**

The online version contains supplementary material available at 10.1186/s12909-022-03270-5.

## Background

There is a growing body of evidence demonstrating the use of digital simulations and games as a training methodology, including use for maternal and child health [1–4]. Multimedia and digital tools can be used as a complement to didactic education to reinforce learning outcomes [5]. Additionally, researchers found that simulation aids assist with supplemental training in nursing education [6]. Another study showed extended realities, including simulation-based realities, to be equivalent to traditional methods but useful in settings where cost is a limitation [7]. Thus, simulation tools have the potential to be an effective education methodology in low-resource settings.

Since 2014, a consortium of organizations, including clinical educators, researchers and simulation experts have collaborated with CARE India and the Government of Bihar, India (GOB) to integrate simulations as in-service training in a large-scale nurse mentoring program focused on maternal and neonatal morbidity and mortality. The largely rural state of Bihar, India has approximately 10% of the country’s population but 15% of all neonatal deaths [8, 9]. The neonatal and maternal mortality rates are disproportionately higher in Bihar than nationally at 34.5 per 1,000 and 165 per 100,000 live births respectively [10–12]. This is in comparison to 24.9 per 1,000 and 122 per 100,000 live births respectively in India overall [10–12]. Furthermore, over the last two decades the proportion of facility-based births in Bihar has been increasing with 76.2% of births occurring institutionally (56.9% in public facilities) [10]. To improve mortality during the time of labor, it is necessary to address the quality of care received at facilities like Primary Health Centers (PHCs) and District Hospitals [13]. With the aim of improving the quality of maternal and neonatal care in Bihar, CARE India integrated PRONTO International’s simulation and team training activities in a nurse mentoring program known as AMANAT (Aapatkalin Matritva Evam Navjaat Tatparta, meaning readiness for emergency obstetric and neonatal care in Hindi). In this program, Nurse Mentor Supervisors (NMS), or expert simulation educators, train Nurse Mentors (NM) in a Train-the-trainer (ToT) mentoring model to conduct simulation exercises in public health facilities across Bihar.

Together with the next phase of the program known as AMANAT Jyoti, the AMANAT nurse mentoring model has proven effective at improving skills, knowledge, and teamwork of nurses in rural Bihar thus far [14, 15]. However, the current challenge of the ToT program functioning at scale is how to sustain the simulation educator skills and knowledge of NMS and NM and maintain engagement of NM working in dispersed clinics across the state. Multiple studies have described a need for refresher trainings in ToT models as knowledge and skills decay over time; and yet, refresher trainings can be timely and resource intensive [16–20]. Refresher trainings require support from local training personnel, added curriculum development, and logistical resources for trainings, which can limit the sustainability of a large nurse mentoring program like AMANAT Jyoti [16, 18]. To address this challenge, digital remote training tools may be an effective strategy to maintain simulation educator skills and engagement in a low-resource setting.

The LIFT Simulation Design Lab at the University of Utah in collaboration with PRONTO International and PRONTO India and with input from the University of California San Francisco (UCSF) developed an interactive video module known as Super Divya to provide simulation educator refresher training and encourage long-term engagement of nurses in AMANAT Jyoti. Following introduction of Super Divya Module 1: Origin Story for NMS in May 2020, this qualitative study aimed to understand the perspectives about, reaction to, and potential impact of Super Divya to overcome the challenges of providing remote support at a large scale to NM educators, including understanding potential to (1) improve skills and competencies of simulation educators, and (2) sustain motivation of nurses in the nurse mentoring program.

## Methods

### Super Divya intervention

As of May 2020, over 720 Nurse Mentors (NM) have been trained in the AMANAT program. In AMANAT Jyoti, the latest phase of the program, this expanded to include 120 Nurse Mentor Supervisors (NMS) trained by CARE India and PRONTO International to become facility-based simulation educators (Table 1). NMS are responsible for educating NM on mentoring activities, including how to conduct simulation and team training activities in health facilities across Bihar in the ToT model.

**Table 1:** CARE India’s Nurse Mentoring Model

*Type of Nurse in AMANAT Jyoti Program*	*Role*	*Training*	*Education Level*
Nurse Mentor Supervisors (NMS) (*n* = 120)	Teach and coach Nurse Mentors how to conduct simulations in facilities	• 5-day SFT^a^ • 4-day ASFT^b^	Post-diploma in nursing, Bachelor’s in nursing, Master’s in nursing
Nurse Mentors (NM) (*n* = 720)	Conduct simulations to enhance teaching of clinical skills to Nurse Mentees in facilities	• 4-day SFT^a ^batch 1 and 2 • 4-day ASFT^b^	General Nursing and Midwifery, Auxiliary Nursing and Midwifery
Nurse Mentees (*n* = 3,000)	Participate in simulations as part of program to strengthen emergency obstetric clinical skills and management	• AMANAT Jyoti training^c^: Jyoti modules 1–4 with 3 days of training per module	General Nursing and Midwifery, Auxiliary Nursing and Midwifery

^a^Simulation Facilitator Training: introduction to PRONTO simulation and team training for educators

^b^Advanced Simulation Facilitator Training: introduction to advanced facilitation and debriefing skills for educators

^c^AMANAT Jyoti training: in-depth simulations and team training modules at facilities

**Fig. 1:**
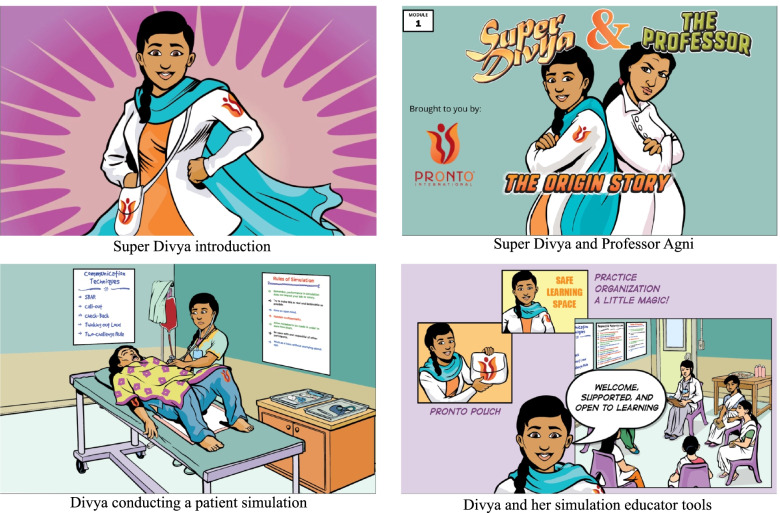
Super Divya: Origin Story

Super Divya is a virtual interactive video module based on a comic superhero protagonist named Divya that aims to reflect the work and cultural reality of NM, reinforce simulation educator skills, and sustain engagement in using simulations in healthcare facilities (Fig. 1). To improve medical education, the use and effectiveness of comics has been well documented in the literature. Maternal and child health comics were cited as an important and most commonly read topic in comic form among medical students in a study in India [21]. Additionally, comics assist with engaging learners, inspiring empathy, and with critical thinking for a deeper understanding of topics in a realistic medical setting [22–24]. The Super Divya introductory module titled ‘Origin Story’ shows Divya using simulation educator skills taught in PRONTO trainings and fighting against the evil antagonist Professor Agni, who is stuck in didactic modes of teaching. Super Divya characters are modeled after NM in Bihar, with similar facility layouts, clothing, accents, and language (Hindi) to engage learners and simulate known medical settings.

The module titled ‘Origin Story’ covers the role of a simulation educator and aspects to remember for conducting high-quality simulation training. The learning objectives are: 1) to recognize elements of positive simulation facilitation, 2) to recognize elements of a poor simulation facilitation, 3) to introduce the characters in Super Divya, and 4) to introduce the PRONTO pouch (the virtual toolkit of a simulation educator). The module was distributed to NMS in May of 2020 via a link on WhatsApp®. Internet connectivity was required to access the module.

### Study design

This study used a framework analysis and idiographic research approach to conduct in-depth, qualitative interviews with NMS [25]. This allowed for flexibility in data analysis and inductive derivation of new themes that were directly related to research aims and arose from the use of a semi-structured interview guide to understand NMS perceptions and attitudes about Super Divya Module 1. Interviews were conducted between May 14, 2020, and June 15, 2020, during the COVID-19 pandemic lockdown. During this period, all in-person PRONTO simulation trainings and activities were paused to prioritize COVID-19 shifts. Interviews were conducted online via Zoom™ from a private location of the participant’s choice instead of in-person to adhere to local policies regarding social distancing and travel.

The principal researcher in this study, who was responsible for all data collected, is Indian by origin and speaks Hindi. The researcher conducted all interviews in the language of the participant’s choice without use of a translator. The researcher did not interact with participants ahead of the study. Instead, participants received verbal information in Hindi and/or English including the purpose of the study, the background of the researcher, and information about the interview process from a research assistant during recruitment (described below). The research assistant, who has an established relationship supervising NMS, was present on the Zoom™ call for about half of the interviews in addition to the researcher. All calls were recorded.

### Sample selection

The population of interest was the subset of 120 NMS that were trained as simulation educators during the AMANAT Jyoti program, with the goal of recruiting at least 20 NMS for interviews to ensure richness of data collection. A list of all NMS working in Bihar was obtained with the help of CARE India staff. Next, participants were recruited based on the following inclusion criteria: currently working as an NMS, received training via SFT or ASFT (Table 1) at least once, and 18 years or older. Using convenience sampling techniques, 22 NMS were selected for interviews with the expectation that some would decline to participate or not answer the phone [26]. The research assistant contacted selected NMS with a request to participate in an interview using a standardized recruitment script (see Additional Files 1).

### Ethical considerations

This study was approved by the ethical review board at the University of California San Francisco (IRB # 20–29902). As all communications were conducted virtually due to the COVID-19 pandemic, verbal informed consent was obtained from each NMS recruited into the study (see Additional Files 2). This included consent to participate, record, and store interview transcripts and consent for publication. The verbal consent procedure was approved by the ethics committee at the University of California San Francisco (IRB # 20–29902). Each participant was assigned a study ID number before the interview began and all interview data were de-identified. Interview notes and recordings were saved on an encrypted, password protected computer and no hardcopies were made.

### Data collection and analysis

Data were collected via semi-structured, in-depth interviews from May 14, 2020-June 15, 2020. The interview guide (see Additional Files 2) consisted of open-ended questions, with room for flexibility to ask additional probing questions based on participant response. The interview guide was pilot tested via Zoom™ with the research assistant in Bihar to ensure the questions were relevant, understandable, and culturally appropriate. The guide was then revised based on pilot interview responses and was translated into Hindi before beginning interviews.

Interviews were conducted in the language of the participants’ choice in either English or Hindi. Data were transcribed within 48 h of each interview. When the interview was conducted in Hindi, it was transcribed and translated directly into English. Interviews continued with participants until thematic saturation was reached, or the point when concepts were repeated by participants and no new data were collected [27]. As interviews were transcribed, they were coded openly using emergent codes from the data using the qualitative analysis tool Dedoose® (see Additional Files 3 for codebook) [28]. We followed Gale et al.'s methodology for synthesis and framework analysis of the qualitative data [25]. Per thematic methodology, codes were grouped based on commonalities into a framework table and overarching themes across interviews were identified. Interviews were re-coded a few months later to ensure validity of analysis.

## Results

Of the 22 NMS conveniently sampled for interviews, two did not respond. Overall, 20 NMS were recruited and provided verbal consent, and 17 interviews were conducted before thematic saturation was reached. On average, interviews took approximately 45 min (range: 23–53 min). Five interviews were conducted in English, seven in Hindi, and five in a mix of the two languages. For two interviews, NMS participated from a clinical facility. NMS self-reported a broad range of experience in nursing, with an average of two years and ten months (range of 1–6 years) (Table 2). However, most participants reported one year or less experience as an NMS (76%, *n* = 13), with an average of one year and three months (range 9 months-2 years and 6 months.). Participants reported attending on average two PRONTO trainings (range of 1–7) with 35% (*n* = 6) attending one and 18% (*n* = 3) attending three or more. Most NMS (71%, *n* = 12) recounted conducting more than ten facility-based simulation trainings and a few (18%, *n* = 3) conducted five or less simulation trainings. Furthermore, the majority of NMS (82%, *n* = 14) recalled viewing the Super Divya module twice or more (range of 1–6), all of whom reported less than one year experience as an NMS. A small number (18%, *n* = 3) reported more than four years’ experience as a nurse, more than 1 year and 6 months as an NMS, recalled attending at least two PRONTO trainings, and remembered conducting more than 20 simulation trainings each.

**Table 2:** Key Characteristics of Nurse Mentor Supervisors (N=17)

	N=17	%	
**No. of** **Super Divya** **viewings**			
Once*	3	*18*	
Twice	7	*41*	
Thrice	3	*18*	
Four or more	4	*24*	
**Years spent as a nurse**			
< 2	5	*29*	
2—< 3	4	*24*	
3—< 4	4	*24*	
≥ 4	4	*24*	
**Years spent as a Nurse Mentor Supervisor**			
< 1	3	*18*	
1—< 2	11	*65*	
> 2	3	*18*	
**PRONTO trainings attended**			
One	6	*35*	
Two	8	*47*	
Three or more	3	*18*	
**PRONTO simulation sessions facilitated**			
< 10	5	*29*	
11—20	4	*24*	
> 20	8	*47*	

^*^Watched Super Divya at least once but could not recall exact number of viewings

Interviews were coded inductively into five broad themes: reactions to Super Divya, Super Divya as a learning tool, accessibility of video platform, relevance for NM, and ideas for further videos.

### Reactions to Super Divya

The majority of NMS said Super Divya modules were entertaining and innovative, with a captivating storyline that maintained interest. Participants found the animation fun and attractive, which added to the videos’ overall approachability.

*“So, when I saw it for the first-time ma’am…I was very meaning curious to know what would happen next…Because the story was narrated so well! So, I was very curious at that time and not at all bored.” (Participant 9, 1 year as a NMS)*

*“Uh, especially animations because I love animations. And it’s sometimes like very…enjoyable to watch animation…like people are talking and playing, um, or role playing or something. Uh, I think animation is a very good part of that video." (Participant 2, 1 year as a NMS)*

Additionally, NMS stated that Super Divya was easy to understand and learn from as it had a simple storyline in Hindi.

*“Ma’am, more videos like this should be made, because these videos ma’am the first thing, the way that they talk in it, so the nurses here, if we show them the video, then it’s such easy language being used, and they talk so well that it’s a story form and the nurses can be taught very well with it.” (Participant 8, 1-2 years’ as a NMS)*

Finally, the character of Divya and her experiences were relatable to NMS experiences, particularly dealing with the effects of negativity and supportive environments on training.

*“And we also relate, no? The same scenario we also, uh, we also have come across when we, when we used to facilitate. So, this type of scenario its really, uh, helpful for us and I really enjoyed that…Because I really related the, uh videos, and uh the real exactly what happened with me when I was facilitating.” (Participant 1,* <*1 year as a NMS)*

### Super Divya as a learning tool

Most participants stated they learned from the Super Divya module. In particular, two thirds said the video taught them about successful simulation facilitation and others mentioned they learned the rules of simulation training. Participants observed the contrast between Super Divya and Professor Agni with positive and negative teaching styles respectively and learned how that impacts learners.

*“And, we got to know overall uh I think the basic first differences between a good facilitator and a bad facilitator…it’s all about the facilitators and how does the simulation have an impact on other people…So, by watching these videos, I, uh…mainly got the idea that if I behave in such and such manner, what impact it will give to my uh person who is performing and how they will react upon it..." (Participant 3,* <*1 year as a NMS)*

*“In this they have shown very well…we* [learners] *behave like this and if someone* [facilitator] *is making us feel comfortable, keeping us well, caring for us and supporting us then we will also think that we should do some good work. But if someone is giving us negativity and just yelling at us each time every time then we will think that oh no, they will just get mad at us again if I do make a mistake…So that I really liked that they differentiated between those two and told us." (Participant 15, 1 year as a NMS)*

Additionally, the vast majority of NMS said Divya taught them how to create a comfortable training space and the qualities required to be a good facilitator, including listening skills, effort, and patience with mistakes.

*"…Super Divya in the video is a very good uh mentor in it. Because she trains her mentees very well, and she gives her hundred percent so, like how a mentor should be uh and how simulations should be run and how things should be utilized and how mentees should be attentive and listen. All those things that are good qualities we see in Super Divya." (Participant 14,* <*1 year as a NMS)*

*“…We have learned not to focus too much on mistakes so that next time they won’t do it wrong, we have to teach them, but not in the way that yeah you were wrong. Later, we need to make them realize that yeah you made a mistake so then they themselves will realize that they made a mistake and next time they won’t do that." (Participant 7, 1 year as a NMS)*

Finally, NMS cited the benefits of visual learning through a video module on memory and recall. They suggested that learning by watching and re-watching videos was more beneficial than learning from one-time, live lectures. They also stated that Super Divya reinforced the value of simulation training more generally as a way to gain clinical skills when risks aren’t as high as they are in a patient interaction.

*“Because I think that a person that looks and sees and listens and learns, they remember that for a long time. In comparison to us going and writing it on the board that these things are there, these are the rules of simulation…people don’t remember as well as they remember from the videos in my opinion." (Participant 8, 1-2 years’ as a NMS)*

*“Well ma’am, it’s the type of video that ma’am without implementing anything on the patient we can practice and learn. Because if we do it on a human body directly and try it, its harmful for the patients also. So, through that video we can gain knowledge where we are lacking and the video is helpful for everyone, not only for the NMS, but for the nurses also. For our nurses, mentors, mentees." (Participant 17, 1 year as a NMS)*

### Accessibility of video platform

When discussing accessibility to Super Divya*,* the majority of NMS responded positively to viewing modules on their phones. Access to strong internet connectivity was cited as an important enabler. Importantly, three participants had to make several attempts or change their phone settings to view the video or were worried about the ease of use for NM who don’t have laptops to access the module and may have trouble with smart phones as well. Two NMS suggested creating an app to access the videos.

*"So, I could uh watch them in my phone I watched on laptop also. So, I did not find any difficulty. Only once I got some difficulty in playing, so but later on I changed the phone setting and it worked. " (Participant 10, 2-3 years’ as a NMS)*

*“Yeah, it’ll be helpful for them* [Nurse Mentors] *but then again we need to make the videos more user friendly for them to understand and to operate. Because as I told you, uh most of them are not even aware as to how to handle Android phones. So, if we are more capable of making them more user friendly and more easy to understand, then I think we, it’ll be much success. Much successful uh version.” (Participant 6, 2-3 years’ as a NMS)*

### Relevance for Nurse Mentors

While the majority of NMS found the videos to be useful for themselves, three participants suggested that NMS have knowledge beyond the module, making it less useful for them as it would be for NM.

*“It can be helpful for us to teach them [Nurse Mentors]. But uh, if in case we want to increase our level of knowledge being a facilitator or something I think we can have something more. But uh if we want to use that particular video to teach our DMTs* [Nurse Mentors] *and mentees then it is good.” (Participant 6, 2-3 years’ as a NMS)*

In fact, all NMS but one agreed that Super Divya videos would be useful for NM to learn how to facilitate simulations. They believed the videos would be enjoyable, relatable, and easy for them to understand.

*“Yeah, uh definitely because…Agni and Super Divya video as…it clearly shows the comparison between good debriefer and uh, I think negative minded debriefer. Because it’s showing them to the mentors…so they will get the idea how to be a good facilitator and debriefer, so distributing it to the AMANAT mentors* [Nurse Mentors] *or even on a further stage to the mentees would definitely be helpful to them to be a good debriefer." (Participant 3,* <*1 year as a NMS)*

*“Yes, it will be helpful for them* [Nurse Mentors] *because these videos that are there, actually here the majority population understands Hindi so, and in this the clear message is given in Hindi itself. And it’s a little animated, so it’s interesting to watch. And it’s not too long too, so it’s not boring…like think as if we are doing simulation tomorrow, then today I will tell them, but if they forget, then they can go home and watch the video and revise. " (Participant 12, 1 year as a NMS)*

Furthermore, participants suggested Super Divya can enable NM to successfully conduct simulation training by helping them prepare. Three NMS mentioned the videos would be most helpful for new Mentors to learn how to conduct simulation training, and four NMS would like the videos to be distributed further to Managers and other staff.

“*Yeah, for uh those DMTs* [Nurse Mentors] *or mentees who are not even aware of the regular things that we work, that we do in labor room, for them it is a good opportunity and good chance to know exactly how to work in an, uh in the same environment that we can create for them. For them it is good, and uh when they first interact with these simulations and debrief, they actually feel that it help them somewhere. Because instead of making those mistakes in real case scenario, they are able to make the mistakes in this real time and then we can rectify the mistakes in our debrief also, so it’s definitely helpful." (Participant 6, 2-3 years’ as a NMS)*

Participants disagreed with one NMS who mentioned Super Divya would be difficult for NM to understand and needs to be changed before distribution.

*“Because, uh, when I saw that video, it was that…uh, it was like that we are trying to give them a comparison between the good person, the good facilitator and the bad facilitator. But I think they need more, something more than that. Because uh what we are facing over here is that uh it’s not about being a good facilitator or bad facilitator, it’s about being a normal facilitator. Because they* [Nurse Mentors] *find it very difficult to being a normal facilitator, let’s forget about good and bad. Its uh, they are not even able to grasp the concept of being a facilitator…So, I think there should be some changes, something very simple that they can understand at their level of knowledge and understanding…I think it’s very important for them to understand their particular role of being a facilitator.” (Participant 6, 2-3 years’ as a NMS)*

In response to this sentiment, in subsequent interviews all NMS disagreed. One stated:

*“No ma’am, actually its animated video, so the* [knowledge] *level of Bihar* [NM] *is not that low that they won’t understand* [smiling and showing levels with her hands] *So, if uh…If they understand our language, right ma’am, then they can understand the videos too." (Participant 17, 1 year as a NMS)*

Additionally, another participant who was worried about accessibility for NM on smart phones suggested translating videos into regional languages to make them more user friendly.

*“Uh, can we, can we make it in uh the regional language that we use over here? Because uh, some of the videos are also provided by GOB, that was uh, that too in their own language, regional languages. I know it is a very difficult task and it, it, it won’t be that much of use because uh Bihar is not only the place where PRONTO is working, but uh if we can try.” (Participant 6, 2-3 years’ as a NMS)*

### Ideas for further modules

When asked what topics they would like to see Super Divya modules address, NMS mentioned wanting to learn both facilitation and clinical skills. About half of NMS want to expand on facilitator skills, including videos of Super Divya facilitating simulation training, how to mentor nurses, communication techniques, and more tips on good facilitation, including behavior, attitude, and creating a comfortable environment. In addition, five NMS mentioned adding videos on how to run debriefing.

*"About behavior meaning uh right now the Super Divya is good, she is cool, and she is taking her uh…sessions in a good way. If the same thing uh we in a better way, well its already very good, but how Super Divya carriers her attitude right, that, how she carries that attitude. Because to be Super Divya you have to have a lot of patience in my opinion right. Explaining to everyone, and acting like a leader, and helping everyone along, that is quite difficult thing so that thing if we, a little bit, uh a little big, if we make it more clear and tell the mentors then they will probably.” (Participant 14,* <*1 year as a NMS)*

Thirteen NMS mentioned wanting videos on clinical skills and complications, such as breech delivery, postpartum hemorrhage, pre-eclampsia/eclampsia, cord prolapse, and more.

*“Like uh right now we have seen different cases like normal delivery, neonatal resuscitation, and postpartum hemorrhage and stuff and how we control it. So, more things like breach, breach delivery and if we do this too then we can make the nurses practice even better…so if these things are added then we can practice even more with this. Like complications-related, meaning these cases don’t always come, they come occasionally, so they aren’t practiced in this. So, these types of things. The other things like neonatal resuscitation, is there, or normal delivery or postpartum hemorrhage, or eclampsia, so these all things meaning, are taught through simulation and they know how to manage it now. So, more things like this, adding complication in simulation, will improve it." (Participant 8, 1-2 years’ as a NMS)*

## Discussion

This qualitative study established that Super Divya virtual education modules have the potential to engage simulation educators to sustain learning and strengthen the capacity of NMS in reinforcing PRONTO simulation training objectives. NMS found Super Divya: Origin Story to be entertaining, approachable, user-friendly, and relatable to their experiences in Bihar. Importantly, they learned important simulation facilitation techniques, including how to create a comfortable environment and the difference between positive and negative simulation facilitation, which shows the potential of Super Divya to improve skills and competencies.

The character of Divya was created to be culturally applicable to the context in Bihar with language and concepts that were matched to the NMS population. Super Divya utilized culturally competent healthcare delivery with culturally specific concepts, linguistic matching, and adaptation of materials to the context in Bihar, India [29]. As Super Divya is specific to this context, NMS found Divya’s experiences to be similar to their own. Relatability of the comics added to the effectiveness of the module. Interestingly, nurses that had spent less than one year in the AMANAT program watched the module more times than those that had more experience. This reinforces the sentiment that Super Divya may be well suited for new simulation educators**.** Additionally, NMS mentioned that the ability to re-watch educational videos is beneficial to learning, which shows that Super Divya can be an effective supplement to in-person training.

Furthermore, all but one NMS reacted positively to distributing Super Divya to NM. This is important, as Super Divya is intended to sustain the facilitation skills and engagement of NM, who are the backbone of the nurse mentoring program but often require additional supervision from NMS. One NMS was especially concerned about accessibility of the videos for NM who may have difficulty with smart phones and language. Additionally, she suggested NM may not be able to understand the simulation facilitation concepts depicted in the video as they do not understand the basics. This was countered, however, by many NMS who were excited about the opportunity to distribute Super Divya to their NM and refuted this opinion.

Moreover, when asked for future topic ideas for Super Divya*,* NMS had various suggestions. More than half of NMS mentioned wanting videos on facilitation skills, including how to conduct debriefing, using communication techniques, and managing behavior and attitude. These suggestions are in line with the purpose of Super Divya and will inform the development of additional modules. Furthermore, three quarters of NMS suggested adding clinical skills and complicated cases, like how to manage postpartum hemorrhage or breech delivery. This shows that NMS may be interested in using the platform of Super Divya to advance knowledge and skills in clinical areas. Animated videos specific to reinforcing obstetric clinical care and skills exist on the SafeDelivery App [30]. NMS have been supplied this app and yet still promoted Super Divya as a tool to help reinforce clinical skills. In addition, there are no tailored virtual education modules to improve the skills and knowledge of simulation educators in literature thus far, rendering the Super Divya tool unique.

Overall, Super Divya is an educational methodology that can be expanded both topically and in scale, with opportunities to disseminate to other mentors, mentees, and staff in Bihar and beyond. This study provided significant lessons learned which have been included in the development of additional modules and also influenced the design of the roll-out of Super Divya to NM*.* We received the feedback that Hindi modules are beneficial for NM, and that NMS liked the character of Divya, the simplicity of the language, the engaging storyline, and the relatability of the videos; all will be included in future modules. Furthermore, NMS requested more modules on facilitation skills as well as clinical skills, which will inform the development of future modules. Teamwork and communication, debriefing, and facilitation tips and tricks were all featured as important topics. Next, one nurse suggested using an app to disseminate modules and a few were worried about accessibility; currently the research team is working on developing and establishing an online Learning Management System to create a permanent home for Super Divya modules.

This study was timely in that interviews began two weeks after distribution of Super Divya: Origin Story. The interview guide was reviewed by PRONTO staff to ensure relevance and understanding. Language concordance was reached with NMS in interviews, although inevitably some meaning may have been lost in translation. Despite the limited generalizability of this study due to the specific, tailored use of interactive video modules for NMS and NM in Bihar, specificity enabled the success of the character Divya. Furthermore, this study demonstrated the ability to utilize virtual education as a means to support NMS in a low-resource setting, including during the COVID-19 pandemic. At a time when all work in the field was halted, pivoting to virtual or hybrid training platforms became more urgent and the innovation of Super Divya allowed for continued engagement with the nurse mentoring and simulation program.

The COVID-19 pandemic also necessitated virtual data collection; interviews were conducted online and were often interrupted and rescheduled due to internet connectivity issues. Furthermore, a number of biases may have been introduced: possible social desirability bias by the presence of a research assistant in interviews, information bias as interviewed NMS were from various districts and clinics with unique work environments and experiences across Bihar, and selection bias as there was no random selection of nurses due to the strict recruitment criteria and high nurse turnover in the program. Finally, due to time and personnel constraints during the pandemic, a secondary review of translated interview transcripts was not conducted.

## Conclusion

Overall, this study demonstrated the positive reactions to and potential usefulness of the specific, tailored, and interactive Super Divya module on Nurse Mentor Supervisors’ engagement and knowledge. The findings from this qualitative study on accessibility and topic areas can inform future modules and implementation strategies. Additional research can help to understand how gains in knowledge from Super Divya translate to improvements in action and behavior related to facilitation skills in the long-term. Given the recent disruption of in-person training by COVID-19, the need for virtual educational tools is critical. As such, Super Divya appears to be an innovative and sustainable intervention that could be easily scalable and has the potential to sustain engagement and knowledge in the Train-the-trainer model nurse mentoring program in Bihar.

## Supplementary Information

**Additional file 1:** Study Recruitment Phone Script.

**Additional file 2:** Provider in-depth-interviews.

**Additional file 3:** Super Divya Qualitative Interviews Framework Analysis Codebook.

## Data Availability

The qualitative data collected and analyzed in this study is not publicly available to protect the anonymity and confidentiality of the participants. Please contact the corresponding author on reasonable request to view de-identified data.

## Abbreviations

AMANAT: Aapatkalin Matritva Evam Navjaat Tatparta (meaning readiness for emergency obstetric and neonatal care in Hindi)
NM: Nurse Mentors
NMS: Nurse Mentor Supervisors
PHCs: Primary Health Centers
ToT: Train-the-trainer model
UCSF: University of California San Francisco
