# Supplementary material for: Super Divya to the rescue! Exploring Nurse Mentor Supervisor perceptions on a digital tool to support learning and engagement for simulation educators in Bihar, India
Source: BMC Med Educ. 2022 Mar 26;22:206. doi: 10.1186/s12909-022-03270-5 (PMC8959557; doi:10.1186/s12909-022-03270-5)
Supplement: Supplementary file 1 — Additional file 1: Study Recruitment Phone Script. [file 12909_2022_3270_MOESM1_ESM.docx]

**Additional File 1**

**Study Recruitment Phone Script**

**Study Team:** Good afternoon, may I please speak with [*name*]?

*If the Person is not available: Thank the person who answered and say goodbye.*

*If the Person is available: First confirm that you are speaking to the correct person.*

**Study Team:** This is Manju Siju calling from PRONTO India in Bihar. I am

a Research Simulation Video Analyst working with Anika Kalra on a research project for PRONTO.

Is this an Ok time for you to speak?

*If the Person says “No” or “I’m not sure”*

**Study Team**: Okay. [*Ask if you can schedule another time to talk. If the person is not sure or seems hesitant, thank him/her and say goodbye.*]

*If the Person says “Yes”*

**Study Team:** Great. I wanted to ask you to participate in a research study about the Super Divya interactive training modules and simulation trainings in Bihar.

Would you like to hear more about this study?

*If the Person says “No” or “I’m not sure”*

**Study Team:** No problem. I wanted to make sure you had the opportunity to learn about the study being done. Thank you for your time.

*If the Person says “Yes”*

**Study Team:** This study is being run by Anika Kalra from the University of California, San Francisco. She is a student helping PRONTO evaluate the Super Divya interactive modules.

We are asking you to be in this study because you are a Nurse Mentor Supervisor who has received the Super Divya training modules and works on simulation training.

We are doing this study because we want to find out your opinions on if the Super Divya interactive training modules are useful for learning about simulation facilitation.

The study includes a Zoom conference call video interview with Anika that I will help you set up. The interview will take place from your home and will take about 45 minutes to an hour to complete. Anika will ask you questions about your opinions and experiences with Super Divya and nurse mentoring.

There will be no direct benefit to you for participating in this study, but what we learn from it may help us improve the Super Divya program in the future. You are allowed to stop the interview at any time and don’t have to answer any questions you do not want to. Additionally, Anika will not save any personal information. She will ask you for permission before recording the conversation. You are allowed to say no.

Being in this study is optional. If you choose to participate, you can tell Anika if you want to stop being in the study at any time. If you choose not to participate, no harm will come to your role as a Supervisor.

**Study Team:** You can contact Anika with any questions you have. You can contact me with any questions too.

Do you have any questions for me at this time?

*Answer any questions patient may have.*

**Study Team:** Would you like to talk with Anika and discuss participating in this study, and if you agree to it, be interviewed?

*If the Person says “Yes” ask for their availability.*

*If the Person says “No” or “I’m not sure”*

**Study Team:** No problem. I will check back in with you in a few days as well. Please feel free to ask us any questions. Anika’s phone number is +1 650 400 8809.

It was nice speaking with you, and we will be in touch. Thank you
