# Supplementary material for: Super Divya to the rescue! Exploring Nurse Mentor Supervisor perceptions on a digital tool to support learning and engagement for simulation educators in Bihar, India
Source: BMC Med Educ. 2022 Mar 26;22:206. doi: 10.1186/s12909-022-03270-5 (PMC8959557; doi:10.1186/s12909-022-03270-5)
Supplement: Supplementary file 2 — Additional file 2: Provider in-depth-interviews. [file 12909_2022_3270_MOESM2_ESM.docx]

**Additional File 2**

Provider in-depth-interviews

DATE____________ RESPONDENT ID____________

Hello, my name is Anika Kalra. I am a Master’s student from the University of California, San Francisco. I am conducting Zoom interviews to learn about your experience as a Nurse Mentor Supervisor.

Anything you tell me will be not be traced back to you, as it will be kept anonymous. This information will be used to understand and improve PRONTO simulation training. The findings may be published to help others who are implementing simulation training in Bihar and beyond.

Since there will be a lot of information that I will not be able to remember or write down, I would like to audio record this interview. Once I transcribe, the audio recording will be deleted. The information you share will NOT have your name or any of your personal identifiers on it.

This interview will take about 45 minutes to complete. There is no right or wrong answer to any of the questions. We want you to speak from your own experiences. There will be time after the interview for you to ask me questions. You can stop the interview at any time, however we encourage you to give your viewpoint on all questions.

Do I have permission to start as well as record this interview?

**SWITCH ON TAPE RECORDER**

**Do I have permission to record this interview?**

**My name is Anika Kalra. I am with respondent number ________ on _____________(date)**

| **Main Questions** | **Specific probes** | **General probes** | **Comments** |
| --- | --- | --- | --- |
| *Ice Breaker Questions* | | | |
| 1. How long have you been a nurse? | Besides your work as a nurse, how do you enjoy spending your time? | 1. Can you tell me more about that? 2. What actually happened? 3. Can you walk me through what happened, step by step? 4. How did that happen 5. Can you give me an example 6. What makes you say that? 7. How did that make you feel? |  |
| 1. How long have you worked as a Nurse Mentor Supervisor? |  |  |  |
| 1. How many PRONTO simulation facilitator trainings have you attended? (i.e. trainings where you were taught how to set up and run simulations) |  |  |  |
| 1. About how many simulation trainings have you facilitated at facilities? (i.e. run and debriefed simulations in a facility) |  |  |  |
| 1. Have you seen the *Super Divya* interactive video modules? | How many modules?  Did you watch the whole video?  Did you watch it more than once?  When did you watch it?  Did you watch or share the video(s) with others? If so, who? |  |  |
| *Perspectives on Super Divya* | | | |
| 1. How did you feel about the *Super Divya* interactive video module(s)? | Was it boring? Entertaining?  Do you want to watch it again?  Share it with others? | 1. Can you tell me more about that? 2. What actually happened? 3. Can you walk me through what happened, step by step? 4. How did that happen? 5. Can you give me an example 6. What makes you say that? 7. How did that make you feel? |  |
| 1. Tell me how you feel about the user friendliness of the Super Divya modules. | Are they easy to use? |  |  |
| 1. How do you feel about the usefulness/ purpose of the Super Divya interactive video module(s)? | Do you find them useful? Why or why not?  Are you aware of the objective of introducing Super Divya video modules as a part of nurse training? |  |  |
| 1. What topics would you like to see *Super Divya* interactive training modules address? | Any other suggestions?  Do you think they help you run simulation trainings in Bihar? |  |  |
| 1. What are your thoughts on distributing *Super Divya* modules to the mentors? | Do you think they would help them run simulation trainings?  Do you think they would find them useful/like them?  Any suggestions for additions or deletions for this population? |  |  |
| *Simulation Facilitation* | | | |
| 1. What does good simulation facilitation mean for you? | What does it entail? | 1. Can you tell me more about that? 2. What actually happened? 3. Can you walk me through what happened, step by step? 4. How did that happen? 5. Can you give me an example 6. What makes you say that? 7. How did that make you feel? |  |
| 1. Tell me about your experience training mentors in simulation facilitation? | *Is it easy for you? Is it hard? Why?*  *What helps you run the trainings?*  *What prevents you from delivering training?*  *Who or what do these barriers come from and why?*  *Are there common areas the DMT Mentors have trouble understanding?* |  |  |
| 1. Tell me about your experience observing the DMT Nurse Mentors? | *What do you gain from this?*  *How necessary do you feel it is for you to observe them?*  *Is it helpful for support?*  *What are some hardships they face and turn to you for help for?*  *Do you have ideas for how they can overcome these hardships without your help?* |  |  |
| 1. Any suggestions for change of the simulation facilitation training component of the nurse mentoring program? | *Should something else be taught?*  *How do you feel about the topics?*  *How do you feel about the usefulness? Why or why not?* |  |  |
| 1. Is there anything else you would like to add? |  |  |  |
| This is the end of the interview. Thank you for your time and help. I am grateful for all the information and ideas you have shared. Do you have any questions for me? |  |  |  |

Summary Impressions
