# Supplementary material for: Super Divya to the rescue! Exploring Nurse Mentor Supervisor perceptions on a digital tool to support learning and engagement for simulation educators in Bihar, India
Source: BMC Med Educ. 2022 Mar 26;22:206. doi: 10.1186/s12909-022-03270-5 (PMC8959557; doi:10.1186/s12909-022-03270-5)
Supplement: Supplementary file 3 — Additional file 3: Super Divya Qualitative Interviews Framework Analysis Codebook. [file 12909_2022_3270_MOESM3_ESM.docx]

**Additional File #3**

Super Divya Qualitative Interviews Framework Analysis Codebook

| **Code** | **Definition** |
| --- | --- |
| User Friendliness | Use for any discussion on accessing and viewing the Super Divya module |
| Entertainment value | Use for any discussion on how entertaining or not entertaining the module was |
| Useful for learning | Use for any discussion on the merits or pitfalls of using Super Divya for learning |
| Easy to understand | Use for any discussion of how relatable or understandable Super Divya is/isn’t |
| Distribution to Nurse Mentors | Use for any discussion of sharing or not sharing Super Divya modules with Nurse Mentors or other staff and why |
| Additions to Super Divya | Use for any discussion on adding/removing topics to Super Divya or future uses for the module and why |
| Enablers for good simulation trainings | Use for any discussion of how Super Divya does/doesn’t help facilitate simulation trainings for Supervisors |
